# Heart failure with preserved ejection fraction: New approaches to diagnosis and management

**DOI:** 10.1002/clc.23321

**Published:** 2019-12-26

**Authors:** Bharathi Upadhya, Dalane W. Kitzman

**Affiliations:** ^1^ Cardiolovascular Medicine Section, Department of Internal Medicine Wake Forest School of Medicine Winston‐Salem North Carolina

**Keywords:** aging, geriatric syndrome, heart failure, preserved ejection fraction, therapy

## Abstract

The majority of older patients who develop heart failure (HF), particularly older women, have a preserved left ventricular ejection fraction (HFpEF). Patients with HFpEF have severe symptoms of exercise intolerance, poor quality‐of‐life, frequent hospitalizations, and increased mortality. The prevalence of HFpEF is increasing and its prognosis is worsening. However, despite its importance, our understanding of the pathophysiology of HFpEF is incomplete, and drug development has proved immensely challenging. Currently, there are no universally accepted therapies that alter the clinical course of HFpEF. Originally viewed as a disorder due solely to abnormalities in left ventricular (LV) diastolic function, our understanding has evolved such that HFpEF is now understood as a systemic syndrome, involving multiple organ systems, likely triggered by inflammation and with an important contribution of aging, lifestyle factors, genetic predisposition, and multiple‐comorbidities, features that are typical of a geriatric syndrome. HFpEF is usually progressive due to complex mechanisms of systemic and cardiac adaptation that vary over time, particularly with aging. In this review, we examine evolving data regarding HFpEF that may help explain past challenges and provide future directions to care patients with this highly prevalent, heterogeneous clinical syndrome.

## 1 INTRODUCTION

Heart failure (HF) with preserved ejection fraction (HFpEF) is the most common form of HF in patients older than 65 years and represents >50% of prevalent HF cases in community.^1^ In the highest age decile, (≥90 years old), nearly all patients with HF have preserved EF. HFpEF is associated with high morbidity and mortality. After HF hospitalization, the 5‐year survival of HFpEF is a dismal 35%, worse than many cancers.^2^ The risk of death in patients with HFpEF increases with increasing comorbidity burden.^3^ Even after adjustment for comorbid conditions, mortality rates associated with HFpEF are higher than in general population age‐matched controls.^4^ Patients with HFpEF have similarly high rehospitalization rates as patients with HF with reduced EF (HFrEF).^5^ In patients hospitalized with HFpEF, 20% are readmitted within 30 days of hospital discharge and >50% within 1 year.^6^ Quality of life in HFpEF is as poor as or worse than HFrEF and is associated with physical activity levels that are as suppressed as those observed in patients with moderate‐to‐severe chronic obstructive pulmonary disease (COPD).^7^ Despite this, there are currently few effective therapies for HFpEF, as most approved therapies for HFrEF have been demonstrated to be ineffective for HFpEF, suggesting major differences in fundamental pathophysiology and therapeutic targets in HFpEF compared to HFrEF. We review relatively recent data that have enhanced our understanding of this complex disorder and that may lead to improved care of patients with this highly prevalent disorder.

## 2 CASE STUDY

A 79‐year‐old woman with long‐standing hypertension, obesity, and type II diabetes presents with shortness of breath on exertion that began 6 months earlier and has since gradually worsened and interferes with daily activities. She denies exertional chest pain. While she is able to shop in the local supermarket, carrying her packages home has become increasingly difficult. She desires to return to her previously active life. Current medications include amlodipine 10 mg daily, metformin 1000 mg daily, Lisinopril 20 mg daily, atorvastatin 20 mg daily. On exam, her blood pressure (BP) is 160/80 mm Hg, heart rate (HR) is 78/minutes, body mass index (BMI) 36 kg/m^2^. She also has peripheral edema; increased jugular venous distention elevated 10 cm above the right atrium. An electrocardiogram did not demonstrate ischemic changes. Her baseline echocardiogram showed mild left ventricular (LV) hypertrophy with an EF of 55% and right ventricular (RV) systolic pressure of 50 mm Hg. During a stress echocardiogram, she exercised for only 3 minutes on a modified Bruce protocol, stopping for extreme shortness of breath. Her resting BP was 160/70 mm Hg and her HR was 76 bpm. At peak exercise, her BP was 196/90 mmHg, with a peak HR of 105 bpm. Echocardiographic images at the end of exercise demonstrated augmentation of contractility of all walls without significant mitral regurgitation. What can be done to improve her symptoms and quality of life?

### 2.1 Making the HFpEF diagnosis: Challenges

Diagnosing HF in older adults poses specific challenges; false‐positive clinical diagnoses are not uncommon.^6^ The most common symptoms of HFpEF are exertional dyspnea. However, symptoms of reduced exercise tolerance are common in the older adults and have been shown to reflect normal physiological changes related to aging or could be related to non‐cardiac etiologies. Furthermore, the diagnosis of HF in the older patients may be difficult due to the presence of co‐morbidities, some of which can mimic HF signs and further confound the diagnosis of HF.^8^ In addition, older patients with HFpEF may not present with “classic” HF symptoms and may instead have very subtle clinical presentations. Up to one‐third of HFpEF outpatients may have a B‐type natriuretic peptide (BNP) level that is below the typical diagnostic thresholds.^8^ This can challenge the common practice of using BNP to make HF diagnosis. In addition, limited predictive capabilities of the echocardiographic variables for diagnosis of diastolic dysfunction further puzzle the clinical picture. There is also no universally agreed upon definition to define HFpEF. The American College of Cardiology/American Heart Association (ACC/AHA) consensus states that the diagnosis of HFpEF is based on typical symptoms and signs of HF in a patient with a normal range LVEF, and no significant valvular abnormalities by echocardiography and no other obvious precipitating factors for HF.^9^ By contrast, the European Society of Cardiology (ESC) requires diastolic dysfunction for the diagnosis of HFpEF, along with symptoms and signs of HF and normal or mildly abnormal LV function.^10^

### 2.2 Aging: A model for HFpEF as a true geriatric syndrome

**Figure 1:**
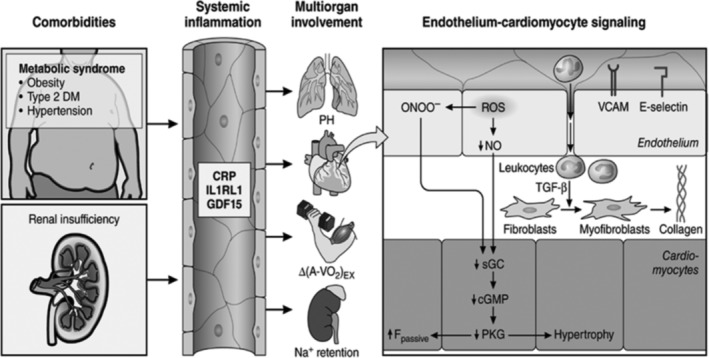
Systemic and myocardial signaling in heart failure (HF) with preserved ejection fraction (HFpEF). Comorbidities induce systemic inflammation, evident from elevated plasma levels of inflammatory biomarkers, such as soluble interleukin 1 receptor‐like 1 (IL1RL1), C‐reactive protein (CRP), and growth differentiation factor 15 (GDF15). Chronic inflammation affects the lungs, myocardium, skeletal muscle, and kidneys leading to diverse HFpEF phenotypes with variable involvement of pulmonary hypertension (PH), myocardial remodeling, deficient skeletal muscle oxygen extraction (ΔA‐Vo2) during exercise (Ex), and renal Na + retention. Myocardial remodeling and dysfunction begin with coronary endothelial microvascular inflammation manifest from endothelial expression of adhesion molecules, such as vascular cell adhesion molecule (VCAM) and E‐Selectin. Expression of adhesion molecules attracts infiltrating leukocytes secreting transforming growth factor β (TGF‐β), which converts fibroblasts to myofibroblasts with enhanced interstitial collagen deposition. Endothelial inflammation also results in the presence of reactive oxygen species (ROS), reduced nitric oxide (NO) bioavailability, and production of peroxynitrite (ONOO^–^). This reduces soluble guanylate cyclase (sGC) activity, cyclic guanosine monophosphate (cGMP) content, and the favorable effects of protein kinase G (PKG) on cardiomyocyte stiffness and hypertrophy. HFpEF indicates heart failure with preserved ejection fraction. (Reproduced with permission from Reference 16)

Cardiac aging is known to affect many, if not all, of the pathophysiological components present in HFpEF. Specific alterations in structural and function in aging, such as ventricular vascular stiffening, vascular dysfunction, impaired [Ca^2+^]_i_ regulation, decreased β‐adrenergic reserve, and physical deconditioning, have been identified as important contributing causes for HFpEF.^11^ Aging is also associated with a decline in a variety of neural, hormonal, and environmental trophic signals; this can leads to loss of muscle mass and mass‐specific strength, characteristic changes in body composition, including decreases in lean body mass and muscle strength, and increases in adiposity which increase vulnerability for sarcopenic obesity.^12^ In addition, older adults hospitalized with a primary diagnosis of HF often have multiple non‐cardiac comorbidities (5.5 on average) and high proportions are frail.^13^ The adverse impacts of aging, frailty and comorbidities on functional capacity and clinical outcomes are cumulative and synergistic.^14^ Indeed, approximately 85% of elderly HFpEF patients are overweight or obese, and the HFpEF epidemic has largely paralleled the obesity epidemic.^15^ Furthermore, aging and obesity are well established risk factors for both HFpEF. Along with comorbidities, aging may initiate and/or aggravate chronic systemic inflammation that may affect myocardial remodeling and dysfunction in HFpEF through a signaling cascade, which begins with coronary microvascular endothelial dysfunction as shown in Figure 1.^3^, ^17^

### 2.3 Key knowledge gap

1. Is HFpEF is a single entity or comprised of several different diseases?
2. Are there inflammatory biomarkers that may help to diagnose HFpEF and better understand its pathophysiology?

### 2.4 Do any meds improve outcomes in HFpEF?

Tables 1 and 2 shows non‐pharmacological and pharmacological clinical trials that were positive in HFpEF on their primary endpoints. Not shown here are the trials that were negative or neutral, which are far greater in number. However, unlike HFrEF, there are currently no disease‐modifying agents available for HFpEF that improve clinical outcomes. Indeed recently, Sacubitril‐valsartan did not result in a significantly lower rate of total hospitalizations for HF and death among patients with HFpEF.^44^ This leads to the question: Why have most pharmacological therapies to date not shown clear benefit in HFpEF? To date, pharmacologic interventions applied in HFpEF have been principally based upon the assumption of underlying, severe neurohormonal abnormalities. However, neurohormonal derangements appear more limited in breadth and severity in HFpEF than in HFrEF. Furthermore, diagnosis of HFpEF is challenging due to the lack of a single objective marker that defines the syndrome, such as a reduced LVEF in HFrEF and the high frequency of comorbidities that may mimic or accompany the HFpEF syndrome. In addition, exercise intolerance, the cardinal manifestation of HF regardless of EF, has a complex pathophysiology and is rarely explained by a single process. Furthermore, most HFpEF studies have only measured diastolic function at rest rather than during exercise where symptoms become manifest.^45^, ^46^ So far, clinical trials generally enrolled “all comers” with clinical syndrome of HF and objective evidence of preserved LVEF. However, evolving evidence indicates that HFpEF is a much more complex disorder than originally thought, influenced by aging processes as explained before, likely systemic in nature, involving many organs and organ systems in addition to the heart, and also involving abnormalities in vascular and skeletal muscle function as well, and likely has multiple phenotypes. These issues and concepts have generally not been addressed in trial designs to date. Given such a multi‐factorial, complex milieu, it is not surprising that drugs and interventions aimed primarily at a central hemodynamics repeatedly failed to strongly impact overall outcomes in HFpEF. As discussed in more detail below, lifestyle modifications (exercise and diet) have been more consistently successful, likely due to addressing HFpEF as a systemic syndrome, and by addressing peripheral, non‐cardiac factors that appear more mutable than cardiac factors.

**Table 1:** Non‐pharmacological interventions that were positive in HFpEF on their primary endpoints

Intervention first author/trial (Ref. #)	HFpEF patient type	Outcomes
Calorie restriction exercise training		Exercise capacity and QOL
SECRET‐1/Kitzman et al^18^ (n = 100)	Ambulatory, stable, obese HF patients (body mass index of 39) with NYHA classes II‐III symptoms (aged 67 ± 5 years, 41% female)	Robust increase in exercise capacity. QOL scores was improved, and benefit was greatest for calorie restriction
Exercise training		Exercise capacity and QOL
PARIS/Kitzman et al^19^ (n = 53)	Ambulatory, stable HF patients with NYHA classes II‐III symptoms (aged 70 ± 6 years, 87% female)	Improved peak and submaximal exercise capacity
PARIS‐II/Kitzman et al^20^ (n = 63)	Ambulatory, stable HF patients with NYHA classes II‐III symptoms (aged 70 ± 7 years, 76% female)	Improved peak VO_2_ and 6MWD
Ex‐DHF trial^21^ (n = 64)	Ambulatory, symptomatic NYHA II/III symptoms, ECHO‐DD (aged 65 ± 7 years, 56% female)	Improved exercise capacity and QOL scores
Smart et al^22^ (n = 25)	Ambulatory, well‐compensated HF (aged 64 ± 8 years, 48% female)	Improved peak VO_2_
Fu et al^23^ (n = 30)	NYHA class II/III HF with episodes of acute pulmonary edema (aged 61 ± 3 years, 33% female)	Improved Peak VO_2,_ diastolic function with reduction of the E/e′ ratio and QOL scores
Gary et al^24^ (n = 32)	NYHA class II/III diastolic HF, h/o ECHO –DD or diastolic HF (aged 67 ± 11 years, all females)	Improved 6MWD, QOL and depression scores
Angadi et al^25^ (n = 9)	NYHA class II/III HF, ECHO‐DD (aged 69 ± 6 years, 11% female)	Improved peak VO_2_ and diastolic dysfunction
Alves et al,^26^ (n = 31)	Admission with clinical signs of HF (aged 63 ± 11 years, 29% female)	Improved exercise tolerance, cardiac systolic (LVEF)and diastolic function (E/e′)
CardioMEMs sensor		Hospitalization for HF
CHAMPION^27^ (n = 119, had LVEF ≥40%	NYHA class III symptoms, hospitalization for HF in last 12 months (aged 62 ± 13 years, 29%female)	Significant and large reduction in hospitalization for patients with NYHA class III HF
Transcatheter interatrial shunt device		LV filling pressure
REDUCE LAP‐HF^28^ (n = 68)	NYHA class II/IV symptoms, PCWP at rest >15 mm of Hg and during exercise >25 mm of Hg measured invasively, (aged 69 ± 8 years, 61% female)	Reduced PCWP during exercise
REDUCE LAP –HF^29^ (n = 44)	NYHA class II/IV, PCWP at rest >15 mm Hg and during exercise >25 mm Hg measured invasively, LVEF >40% (aged 70 ± 9 years,50% female)	Reduced PCWP during exercise
Adaptive servo‐ventilation	Moderate to severe sleep disorder breathing	NYHA class, LV diastolic function, CV hospitalization
Yoshihisa et al^30^ (n = 36)	NYHA class II‐IV HF, stable clinical status, with moderate to severe sleep disorder breathing (age ± 16 years, 11%female)	Improved NYHA class, LA volume, BNP, ECHO‐DD, Proportion of patients had less CV events or hospitalization for HF
CAT HF^31^ (n = 126)	Hospitalized HF, BNP ≥300 pg/mL, with moderate to severe sleep apnea, 24 (19%) had HFpEF (aged 61 ± 14 years, 26% female)	The risk of the primary composite endpoint was reduced by 62% Composite global rank score (hierarchy of death, CV hospitalizations, and percent changes in 6MWD)

Abbreviations: A‐VO_2_ Diff, arterial‐venous oxygen difference; CV, cardiovascular; DD, diastolic dysfunction; ECHO, echocardiographicaly assessed; E, mitral early diastolic velocity; e′, mitral annular velocity; HF, heart failure; HFpEF, heart failure with preserved ejection fraction; LA, left atrium; LV, left ventricle; MWD, minute walk distance; n, number of participants; NYHA, New York Heart Association; QOL, quality of life; PCWP, pulmonary capillary wedge pressure; VO_2_, oxygen consumption; B‐type natriuretic peptide.

**Table 2:** Pharmacological interventions that were positive in HFpEF on their primary endpoints

Intervention first author/trial (Ref.#)	HFpEF patient type	Primary outcomes
ACE‐I/ARB		CV death/HF admissions
CHARM‐Preserved^32^/Candesartan (n = 3023)^a^	NYHA classes II‐IV HF with prior cardiac hospitalization (aged 67 ± 11 years, 40% female)	Fewer HF admissions
The PEP‐CHF^33^/Perindopril (n = 852)^a^	Diagnosis of HF and treated with diuretics and an ECHO‐DD. Prior cardiac hospitalization within 6 months. (aged 76 ± 5 years, 55% female)	Fewer HF admissions, improved symptoms and exercise capacity
Aldosterone antagonists		HF admission, LV remodeling and LV filling pressure
TOPCAT^34^/Spironolactone (n = 3445)^a^	Patients had h/o HF hospitalization within previous 12 months and elevated BNP within 60 days before randomization. (aged 69 years [median], 52% female)	Modest decline HF hospitalization
Aldo‐DHF^35^/Spironolactone (n = 422)	Ambulatory patients/NYHA class II‐III symptoms, ECHO‐DD and normal or near‐normal BNP levels. (aged 67 ± 8 years, 52% female)	LV remodeling, neurohumoral activation were improved
Kosmala,et al^36^/Spironolactone (n = 150)	NYHA class II/III, ECHO‐DD, and baseline increased exercise E/e′ ratio. (aged 67 ± 9 years; 85% female)	Improvement in exercise capacity. Reduction in exercise‐induced ECHO measure of increased LV filling pressure
Inorganic nitrates		Exercise capacity, Biventricular filling and pulmonary pressure
Borlaug et al^37^/Inhaled sodium nitrite (n = 26)	Elevated PCWP at rest (>15 mmHg) and with exercise (≥25 mmHg). (aged 70 ± 9 years, 54% female)	Acute administration reduced biventricular filling pressures and PAP at rest and during exercise
Kitzman et al^38^/ Beet root juice (n = 20	Ambulatory HF patients with NYHA classes II‐III (aged 69 ± 7 years of age)	Improved submaximal Endurance
Zamani P et al^39^/NO3‐rich beetroot juice (n = 17)	Symptomatic HF, ECHO‐DD, elevated NT‐pro‐BNP or PCWP >12 mmHg on prior cardiac catheterization. (aged 66 ± 9, 12% female)	Improved peak VO_2_ in subjects with HFpEF by significant reduction in systemic vascular resistance
Phosphodiesterase‐5 inhibitor		PAP and RV function
Guazzi, et al^40^, ^41^/Sildenafil (n = 44)	HF signs and symptoms, ECHO‐DD, invasively measured PASP >40 mmHg. (aged 72 years [median], 20% female)	Improvement in PAP, RV function and dimension, LV ventricular relaxation and distensibility
Vericiguat (soluble guanylate cyclase stimulator)^a^		Change in NT–proBNP and LA volume index
SOCRATES‐PRESERVED^42^ (n = 477)	73 ± 10, 48% female, NYHA class II‐IV, LVEF ≥ 45%, increased BNP ≥ 100 pg/mL or NT‐proBNP levels ≥ 300 pg/ML, ECHO evidence of DD, LVEF ≥ 45%.	Improvements in quality of life
LCZ696(ARNI)^b^		NT‐proBNP
(Sacubitril/valsartan)		
PARAMOUNT^43^/(n = 301)	NYHA class II‐III HF, NT‐pro BNP > 400 pg/nL and be on a diuretic therapy. (aged 71 ± 9 years, 57% female)	Significant reduction in NT‐proBNP

Abbreviations: ACEI, angiotensin‐converting enzyme inhibitors; ARBs, angiotensin receptor blockers; ARNI , angiotensin receptor‐neprilysin; BNP, B‐type natriuretic peptide; DD, diastolic dysfunction; E, Mitral early diastolic velocity; e′, mitral annular velocity; ECHO , echocardiographicaly assessed; HFpEF, heart failure with preserved ejection fraction; HF, heart failure; LVEF, left ventricular ejection fraction; LA, left atrium; LV, left ventricle; n, number of participants; NT pro BNP; N terminal, B‐type natriuretic peptide; NYHA , New York Heart Association; PCWP, pulmonary capillary wedge pressure; PAP , pulmonary artery pressure; RV , right ventricle.

a Neutral on composite primary outcome.

b Except this trial, among all other trials study drug was compared with placebo. In this trial, comparison made between study drug and valsartan.

### 2.5 Key knowledge gap

1. Was the lack of definite benefits in pharmacological trials to date caused by a flawed study designs or by ineffective study interventions?
2. Should future HFpEF trials include broader group of subjects or individual subpopulations?

### 2.6 Prevent and delay the progression of HFpEF

Table 3 summarizes the practical approaches to managing HFpEF. In older patients, multi‐level strategies and interventions aimed at improving adherence to guidelines and tailoring therapy could be the key to improving outcome, and to reducing costs related to HF‐related re‐admissions. An important component of treating a patient with HFpEF is treating the contributing factors and comorbidities that are frequently present and significantly impact the clinical course, such as obesity, hypertension, coronary artery disease, diabetes, COPD, anemia, chronic kidney disease, and sleep‐disordered breathing.^9^ Several hypertension trials, including the systolic BP intervention trial (SPRINT), have shown a reduction in incident HF with treatment of hypertension, although these trials did not differentiate between incident HFpEF and HFrEF.^47^, ^48^, ^49^ Considering the age distribution in these trials and the age‐dependent relative incidence of HFpEF, control of hypertension may be the single most important prevention strategy for HFpEF. In SPRINT, both HFpEF and HFrEF incident cases were significantly reduced, including specifically in older patients ≥75 years old.^50^ The BP goals in the ACC/AHA HF guideline are similar to those in the general population, with the exception that the 2017 ACC/AHA HF guideline update recommends the lower systolic BP target of 130 mm Hg.^9^, ^48^, ^51^ ACC/AHA HF guidelines support the use of beta‐blockers, angiotensin‐converting enzyme inhibitors (ACEI), and angiotensin receptor blockers (ARBs) for hypertension (IIa recommendation) and ARBs and aldosterone antagonists receive a relatively weak recommendation (class IIb, level of evidence B) as reasonable to consider for decreasing hospitalizations in HFpEF. *Metabolic risk factors*: HFpEF patients demonstrate a high prevalence of obesity and diabetes. Increased adiposity promotes inflammation, hypertension, insulin resistance, and dyslipidemia and impairs cardiac, arterial, skeletal muscle, and physical function,^7^, ^52^, ^53^ all of which are common in HFpEF and contribute to its pathophysiology.^54^ Recently, studies in type 2 diabetes patients showed reduced risk of HF hospitalization in patients receiving either empagliflozin or dapagliflozin, which are novel sodium‐glucose cotransporter 2 inhibitors,^55^, ^56^ *Coronary Artery Disease (CAD)* Patients with HFpEF and symptoms and signs of ischemia are treated with standard therapy including beta‐blockers and calcium channel blockers.^57^ Patients with epicardial CAD may require complete coronary revascularization by percutaneous coronary intervention or coronary artery bypass graft surgery.^57^ However, retrospective data suggest that clinically evident, acute coronary ischemia may not be the key trigger for acute decompensation in HFpEF, that the EF does not decline during an acute episode,^58^ and that revascularizing epicardial coronary stenoses has little effect on preventing the recurrence of acute HFpEF.^59^ *Atrial fibrillation (AF)* prevalence has been increasing due to an aging general population and increased longevity. AF in HFpEF associated with impaired LV systolic, diastolic function and functional reserve, larger left atria (LA) with poor LA function, RV dysfunction, more severe neurohumoral activation, and impaired exercise tolerance.^60^, ^61^ Tachycardia is also deleterious by shortening the time of diastole that may impair adequate diastolic filling. For these reasons, restoration and maintenance of sinus rhythm are preferred when AF occurs in patients with HFpEF. To restore sinus rhythm, cardioversion is recommended because catheter ablation of AF had limited long‐term success in HFpEF.^62^ If cardioversion is unsuccessful, rate control and permanent anticoagulation become mandatory.^57^ *Anemia* is more prevalent in HFpEF than in HFrEF patients and associated with increased risk of HF hospitalization and overall mortality.^63^ The 2017 ACC/AHA HF management update included a class IIb recommendation for iron replacement therapy in appropriately selected patients, although HFpEF patients have not been included in the cited trials.^9^ Treatment of anemia with erythropoietin analogs received a class III recommendation (no benefit).^9^

**Table 3:** Practical management of heart failure with preserved ejection fraction

Diuretics at the lowest effective dose for signs and symptoms of volume overload
Moderate sodium restriction diet
Every patient should have a home scale, weigh themselves daily, and be provided with instruction for steps to take based on weight changes
Comprehensive HF disease management, including education, close follow‐up, particularly for recently hospitalized patients
Control of blood pressure, diabetes, and other comorbidities
Avoid iatrogenic volume overload
Restoration and maintenance of sinus rhythm, control of heart rate in patients with permanent AF
Search for and treat symptomatic myocardial ischemia
Formal sleep assessment in HF patients with suspicion of sleep disordered breathing or excessive daytime sleepiness
Regular moderate physical activity

Abbreviations: AF, atrial fibrillation; HF, heart failure.

### 2.7 Key knowledge gap

1. Is rate control alone or rhythm control the best strategy for treatment in HFpEF patients?
2. What is the best way to manage comorbidities in HFpEF patients?

### 2.8 Lifestyle interventions in HFpEF

**Figure 2:**
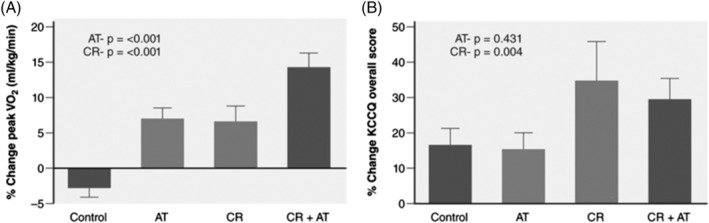
Effects of a 20‐week caloric restriction diet on exercise capacity and quality of life in heart failure (HF) with preserved ejection fraction (HFpEF). The graph displays percent changes ± SEs at the 20‐week follow‐up relative to baseline by randomized group for peak VO2 (mL·kg^–1^·min^–1^, A) and quality of life scores, *P*‐values represent effects for AT and CR. AT indicates aerobic exercise training; and CR, caloric restriction diet. (Reproduced with permission from Reference 16)

Recent data support the beneficial impacts of lifestyle modification, including weight reduction, dietary and nutrient consumption, physical activity, and cardiorespiratory fitness on HF risk. In a pooled analysis of 51 000 participants from the Women's Health Initiative, Multiethnic Study of Atherosclerosis, and Cardiovascular Health Study cohorts, the risk for incident HFpEF increased in a dose‐dependent manner as BMI increased and leisure‐time physical activity declined.^45^ Recently, Kitzman et al showed that among older obese patients with chronic, stable HFpEF, intentional weight loss via calorie restriction (CR) diet significantly improved exercise capacity to a degree similar to and was additive to exercise training (ET).^18^ In addition, CR but not exercise significantly improved the HF specific quality of life measures (Figure 2, Table 1).^18^ Even though, a recent meta‐analysis of randomized trials among older patients without HF indicates that CR is associated with a 15% reduction in total mortality,^64^ because of the reported “HF obesity paradox,” further studies are needed to determine role of CR in older patients with HFpEF.^42^

In 2010, Kitzman et al reported the first randomized controlled trial evaluating ET as a treatment for HFpEF, showing substantial improvement in cardiorespiratory fitness with training (Table 1). 19 Since that time, a number of other studies have substantiated this benefit and demonstrated favorable effects on quality of life (Table 1). Existing data suggest that the majority of ET‐related improvement in exercise capacity may be related to microvascular and/or skeletal muscle adaptations that increase diffusive oxygen transport and/or utilization by the active muscles.^65^, ^66^ A supervised maximal exercise test with monitoring for ischemia should be performed before HFpEF patients beginning an exercise program. Exercise protocols used in clinical trials primarily included aerobic‐type activities, such as walking, stationary cycling, or rowing. After supervised setting with direct supervision and monitoring, depending on individual progress, patients may be able to be transitioned to a home exercise maintenance training program. Randomized exercise intervention trials also showed that ET appeared safe in older, deconditioned HFpEF patients (Table 1), although the trials were not large enough or designed to definitively address the question of safety. For the same reasons, the potential impact of ET in HFpEF on clinical events, including hospitalizations and death, is unknown. Recently, the REHAB‐HF, prospective, multicenter pilot trial which successfully randomized 27 patients ≥60 years of age hospitalized with acute decompensated HF (both HFrEF and HFpEF) showed that a novel, tailored, progressive, multidomain physical rehabilitation is feasible in older patients with acute decompensated HF who have high rates of frailty and comorbidities and has the potential to improve physical function and reduce rehospitalization rates.^67^ A larger trial is underway to verify these findings.

### 2.9 Exercise prescription

A supervised maximal exercise test with monitoring for ischemia should be performed before HFpEF patients beginning an ET program. The ET program for stable HFpEF patients should consist of continuous large muscle mass moderate intensity endurance exercise performed for 20 to 60 minutes per session, 3 to 5 days per week. The exercise is usually performed on a bicycle or treadmill. The duration and frequency of effort should be up titrated before intensity is increased. Once patients demonstrate a tolerance of aerobic training levels, resistance training activities should be considered. It is recommended to initiate ET in a structured, supervised, center‐based program. This can be either in‐hospital or in a specialized facility, as long as close supervision are available. After supervised setting, depending on individual progress, patients usually should be able to be transitioned to a home exercise maintenance training program. Ideally, a patient‐tailored ET program is prescribed instead of a “one size fits all” approach especially in older patients with HFpEF. In addition, to increase long‐term adherence to ET, the patient's preferences should be taken into account.

ACC/AHA guidelines recommend moderate, regular physical activity for all HF patients, which seem reasonable. However, in the absence of data regarding effect of ET on clinical events, the Centers for *Medicare & Medicaid Services* does not reimburse *for cardiac rehabilitation* in either acute or chronic HFpEF patients, in contrast to its policy for chronic (but not acute) HFrEF.

### 2.10 Key knowledge gap

1. What is the most effective and safe exercise prescription for older HFpEF patient?

### 2.11 Treatment of congestion

In the CHAMPION trial (CardioMEMS Heart Sensor Allows Monitoring of Pressure to Improve Outcomes in NYHA Class III HF Patients trial), clinical management guided by physician knowledge of central hemodynamics significantly reduced HF hospitalization (Table 1).^27^, ^68^ This finding was confirmed in more recent analyses of Medicare beneficiaries.^67^ The CARDIOMEMS device is a wireless, implanted pulmonary artery pressure monitor implanted in the distal PA during right heart catheterization. Patients transmit hemodynamic data daily using a wireless RF transmitter.

Given that rises in left atrial (LA) pressure and pulmonary venous congestion are shown to herald HF events in patients with HFpEF, creating a controlled left‐to‐right interatrial shunt to allow LA decompression could be a rational non‐pharmacological strategy for alleviating symptoms. The Reduce Elevated LA Pressure in Patients with HF (REDUCE LAP‐HF) study is a multicenter, prospective, non‐randomized, single‐arm phase 1 study designed to assess the safety and performance of the device in patients with HFpEF with NYHA II‐IV despite optimal medical or device therapy and demonstrated reductions in LA pressure during exercise with improvements in functional capacity and health‐related quality of life scores 6 and 12 months after implantation of this device (Table 2).^69^ Recently, REDUCE LAP‐HF I, a phase 2 randomized parallel‐groups, and blinded multi‐center sham‐controlled trial published short‐term results (Table 2).^29^

### 2.12 Key knowledge gap

1. Can we do remote hemodynamic monitoring effectively in older patients?
2. What are the best metrics for determining when a patient is adequately decongested during an acute decompensated HF hospitalization?

Thinking like a Geriatrician—“Sometimes the disease is not the most important focus—maintaining the patient's function is” as said by Dr. Covinsky.

In older patients hospitalized primarily for HF, many factors outside the heart such as advanced age, globally reduced organ system reserve capacity, physical frailty, impaired cognition, and comorbidities strongly influence outcomes.^70^ In addition, the hospital environment—with immobilization, fasting, sleep deprivation, and disorientation—can dramatically worsen physical frailty with rapid, severe loss of muscle mass and function.^70^ When older HF patients are thought to be ready for discharge, careful attention should focus on their multiple comorbidities, globally reduced organ reserve, severe physical deconditioning, and cognitive dysfunction to prevent the “post‐hospitalization syndrome,” consisting of high rates of rehospitalization, mortality, and nursing home admission, prolonged physical disability, poor quality of life, and high health care costs.^71^ Thus, it is important to treat not just the disease and but the whole patient particularly in the older population. Improved care of complex older patients with HFpEF is dependent on a new model of collaboration and teamwork between primary care provider, geriatrician, and cardiologist, with timely access to palliative care to accommodate the fundamental heterogeneity of aging and the patient's choices. In addition, cardio‐geriatric clinics are designed to meet the needs of older patients with HF and their caregivers by providing comprehensive care focusing on improving quality of life and functional independency. If managed adequately in a multidisciplinary ambulatory care setting, we can potentially prevent most of the unnecessary hospital readmissions. To improve care delivery and minimize the need for emergency visit and rehospitalization, development of ambulatory services with effective chronic disease management and integrated care programs are needed urgently.

### 2.13 Key knowledge gap

1. What are the optimal disease management strategies/programs and transitional care for older hospitalized HFpEF patients?
2. How can we best implement interdisciplinary approaches to treat this unique and rapidly growing population?

### 2.14 HFpEF management based on clinical phenotypes

**Figure 3:**
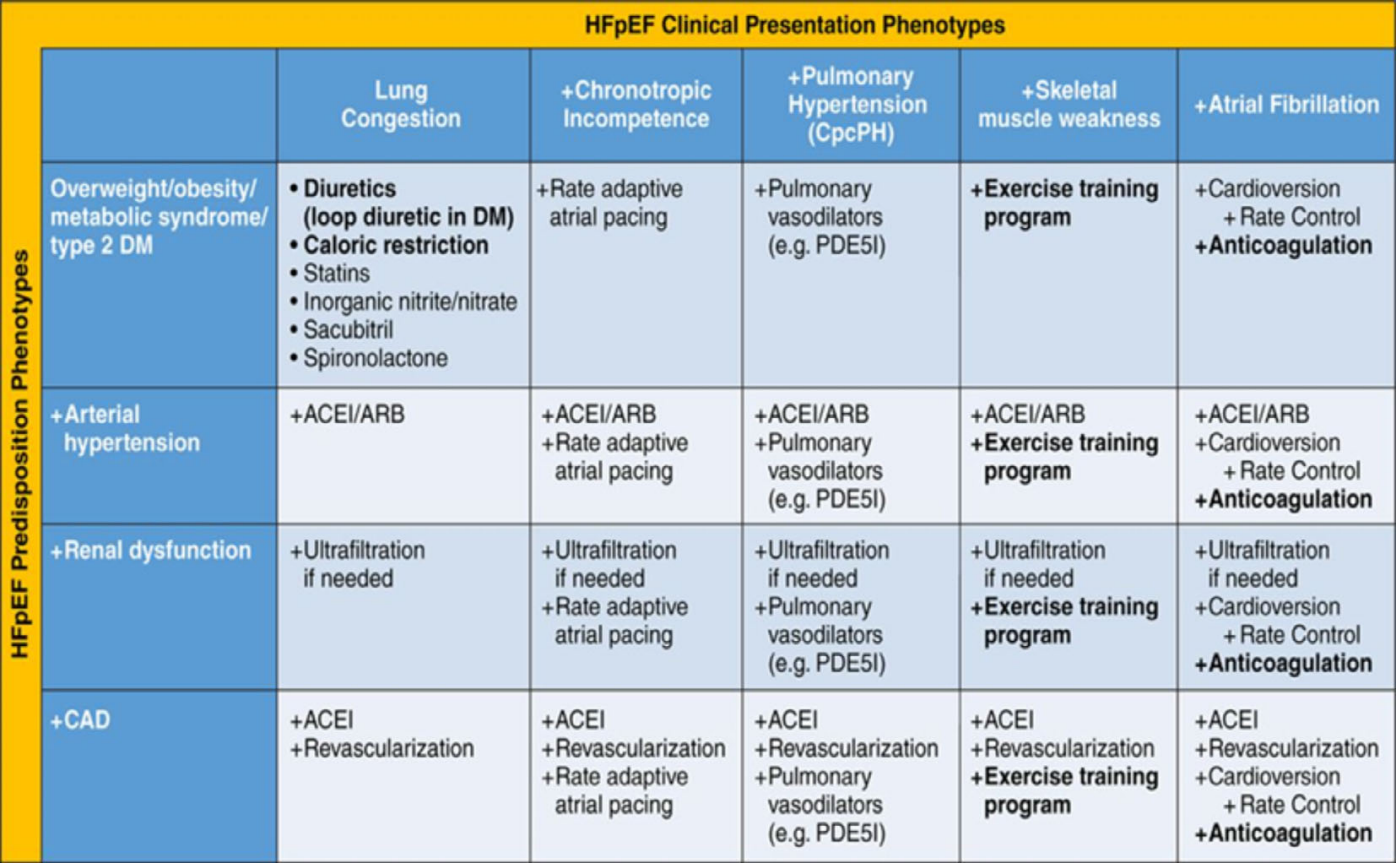
Phenotype‐specific heart failure (HF) with preserved ejection fraction (HFpEF) treatment strategy using a matrix of predisposition phenotypes and clinical presentation phenotypes. (Reproduced with permission from Reference 73)

A key evolving concept in HFpEF therapy is that the disorder is highly heterogeneous and manifestations can vary markedly from patient to patient even within a specific HFpEF patient population.^72^ More recently, Shah et al proposed a matrix combining predisposition phenotypes with clinical presentation phenotypes as a starting point to guide clinical care in HFpEF (Figure 3).^7^ The approach starts with general treatment recommendations beneficial to the majority of HFpEF patients because they address the presentation phenotypes of lung congestion and the most common phenotype of overweight/obesity, present in >80% of HFpEF patients. Subsequently, supplementary recommendations are suggested for additional phenotypes, such as metabolic/obesity, arterial hypertension, renal dysfunction, and coronary artery disease. Additional clinical presentation phenotypes are suggested in whom specific therapeutic interventions could be meaningful like chronotropic incompetence, pulmonary hypertension, skeletal muscle weakness, and AF. This phenotype‐specific approach may prove a valuable advance.

### 2.15 HFpEF management based on biologic phenotypes

An understanding of which are the causes and which are the downstream effects, may allow the HFpEF syndrome to be concentrated into distinct diagnoses based on the underlying biology. From this, specific interventions can follow, targeting individuals identified on the basis of their biological phenotype. This concept was recently expanded upon by Lewis et al,^74^ suggesting that determining the underlying pathobiologic disease mechanism which ultimately leads to specific clinical phenotypes can drive discernment of “biological phenotype” and integrate with the clinical” phenotypes” thereby further enhancing the potential gains from individualized approaches on the basis of enhanced phenotypic categorization. Biological heterogeneity has potentially compromised in prior HFpEF trials, thus HFpEF now needs “to get personal.”

### 2.16 Key knowledge gap

1. Should HFpEF diagnosis and management be clinically or mechanistically based?

### 2.17 Proposals for the future: Clues to be remembered

(a) Diastolic dysfunction by itself is not enough to establish HFpEF. (b) HFpEF is not simply a disease of aging nor does it occur only in females. (c) HFpEF has significant phenotypic and etiologic heterogeneity. (d) Due to its heterogeneity, a “one‐size‐fits‐all” strategy is unlikely to work in HFpEF. (e) HFpEF is associated with multiple comorbidities. (f) HFpEF is a multi‐system disease, with the heart being a major component but with others providing major contributions.^72^ (g) To date, two strategies that have been shown most definitively to be beneficial for improving clinically meaningful outcomes in HFpEF, ET and CR and both of these interventions have broad, pleotropic, systemic, and anti‐inflammatory effects, as well as favorable effects on multiple organ systems, including on arterial, cardiac, and skeletal muscle. (h) Clinical trials defining optimal management for comorbidities have by and large excluded HFpEF patients. In addition, much broader research into myocardial and non‐myocardial abnormalities at a tissue level in carefully phenotyped HFpEF subgroups is very much needed.

### 2.18 Case conclusion

Our 79‐year‐old woman should be placed on diuretics at the lowest effective dose for symptomatic relief. She should have a home scale and weigh herself daily. We should provide instruction for steps to take diuretics based on weight changes. Her BP was not well controlled and lisinopril to be adjusted to keep SBP < 130 mm of Hg. She should be advised regarding dietary compliance. She should be encouraged to exercise daily. She should be provided comprehensive HF disease management, including education, diet, exercise therapy, and close follow‐up. Ideally as described earlier, she is recommended to participate in a structured, supervised ET program; however, lack of CMS coverage can be a major barrier to formal cardiac rehab in older HFpEF patients.

## 3 CONCLUSIONS

HFpEF is the most common form of HF in the community, its prevalence is increasing, and prognosis has not improved or even worsened. It is nearly unique to older adults and is a true geriatric syndrome. Despite a moderate number of clinical trials, therapeutic successes to date have been few, and clinical management is largely empiric. An evolving paradigm suggests that, like other geriatric syndromes, HFpEF is complex and multifactorial, probably systemic, and clinically heterogeneous and has a multifactorial pathophysiology, underlying age‐related changes, frequent multiple chronic comorbidities and multiorgan involvement. Understanding the relationship between HFpEF and aging may help with understanding the biology of HFpEF more generally. In addition, machine learning techniques suggested by Shah et al, when applied to large phenotyped (both biological and clinical) datasets in combination with clinical outcomes may help to improve our understanding of how biological phenotypes integrate with clinical phenotypes. Efforts are underway to utilize these concepts to identify novel therapeutic targets, improve the design of future clinical trials, and to develop effective clinical management algorithms. Finally, the complexities of these patients demand an approach that is more holistic by addressing not only direct HF‐related conditions but also optimal management of geriatric syndromes, focusing on quality of life.

## CONFLICT OF INTEREST

Dr. Kitzman declares the following relationships: Consultant for Abbvie, Bayer, Merck, Medtronic, AstraZeneca, Merck, Corvia Medical, and Actavis, research grant funding from Novartis, Bayer, and AstraZeneca, and stock ownership in Gilead Sciences and Relypsa. Dr. Upadhya has received research funding from Novarits and Corvia.
